# Feasibility of a clinical-radiomics combined model to predict the occurrence of stroke-associated pneumonia

**DOI:** 10.1186/s12883-024-03532-3

**Published:** 2024-01-25

**Authors:** Haowen Luo, Jingyi Li, Yongsen Chen, Bin Wu, Jianmo Liu, Mengqi Han, Yifan Wu, Weijie Jia, Pengfei Yu, Rui Cheng, Xiaoman Wang, Jingyao Ke, Hongfei Xian, Jianglong Tu, Yingping Yi

**Affiliations:** 1 Department of Medical Big Data Research Centre, The Second Affiliated Hospital of Nanchang University, 1MinDe Road, Nanchang, 330006 P.R. China; 2 School of Public Health, Jiangxi Provincial Key Laboratory of Preventive Medicine, Nanchang University, Nanchang, China; 3 Department of Neurology, The Second Affiliated Hospital of Nanchang University, 1MinDe Road, Nanchang, 330006 P.R. China

**Keywords:** Stroke-associated pneumonia, Radiomics, Prediction, Acute ischemic stroke, Magnetic resonance imaging

## Abstract

**Purpose:**

To explore the predictive value of radiomics in predicting stroke-associated pneumonia (SAP) in acute ischemic stroke (AIS) patients and construct a prediction model based on clinical features and DWI-MRI radiomics features.

**Methods:**

Univariate and multivariate logistic regression analyses were used to identify the independent clinical predictors for SAP. Pearson correlation analysis and the least absolute shrinkage and selection operator with ten-fold cross-validation were used to calculate the radiomics score for each feature and identify the predictive radiomics features for SAP. Multivariate logistic regression was used to combine the predictive radiomics features with the independent clinical predictors. The prediction performance of the SAP models was evaluated using receiver operating characteristics (ROC), calibration curves, decision curve analysis, and subgroup analyses.

**Results:**

Triglycerides, the neutrophil-to-lymphocyte ratio, dysphagia, the National Institutes of Health Stroke Scale (NIHSS) score, and internal carotid artery stenosis were identified as clinically independent risk factors for SAP. The radiomics scores in patients with SAP were generally higher than in patients without SAP (*P* < 0. 05). There was a linear positive correlation between radiomics scores and NIHSS scores, as well as between radiomics scores and infarct volume. Infarct volume showed moderate performance in predicting the occurrence of SAP, with an AUC of 0.635. When compared with the other models, the combined prediction model achieved the best area under the ROC (AUC) in both training (AUC = 0.859, 95% CI 0.759–0.936) and validation (AUC = 0.830, 95% CI 0.758–0.896) cohorts (*P* < 0.05). The calibration curves and decision curve analysis further confirmed the clinical value of the nomogram. Subgroup analysis showed that this nomogram had potential generalization ability.

**Conclusion:**

The addition of the radiomics features to the clinical model improved the prediction of SAP in AIS patients, which verified its feasibility.

## Introduction

Stroke-associated pneumonia (SAP) is one of the most common medical complications in patients with acute ischemic stroke (AIS), with an estimated incidence ranging between 5 and 26% [1]. SAP reduces the quality of life and increases the treatment costs, hospital stay, and risk of mortality in AIS patients [2–6]. Therefore, there is a need to develop fast and reliable tools to identify high-risk patients to improve clinical outcomes.

Previous studies have established different scoring systems for early pneumonia prediction after AIS, such as the A2DS2 scale, the AIS-APS scale, and the ISAN scale [7–9]. However, these tools are based solely on clinical data, and their prediction efficiency is moderate [10]. Brain imaging is necessary to diagnose stroke and evaluate the extent of the disease. Diffusion-weighted imaging-magnetic resonance imaging (DWI-MRI) is the most sensitive and accurate imaging method for diagnosing AIS and has been widely used in studies related to stroke [11, 12]. Several studies identified a correlation between the brain infarct size on DWI-MRI and prognosis following an AIS and may also have a role in the development of complications [13–15]. Studies have also found an association between several MRI radiological features, including the location, infarct volume, the number of lobes involved, and the brain atrophy score, with the risk of developing SAP [16, 17].

Radiomics uses algorithms to objectively extract a large number of quantitative features from medical images. This data can be used to transform subjective visual evaluation into an objective evaluation data-driven evaluation of traditional radiologic characteristics [18–22]. This technique is increasingly being used to facilitate the diagnosis of stroke lesions [23, 24], predict early outcomes [25–27], and evaluate the long-term prognosis of stroke [28, 29]. However, to our knowledge, no studies have been conducted evaluating the role of radiomics in predicting SAP following an AIS.

Therefore, in this study, we aimed to explore the predictive value of radiomics in predicting SAP and construct a prediction model based on clinical features and DWI-MRI radiomics features to predict SAP following AIS. The model was developed into a nomogram, and decision curve analysis (DCA) was performed to evaluate the clinical utility of the model.

## Materials and methods

### Study population

**Fig. 1:**
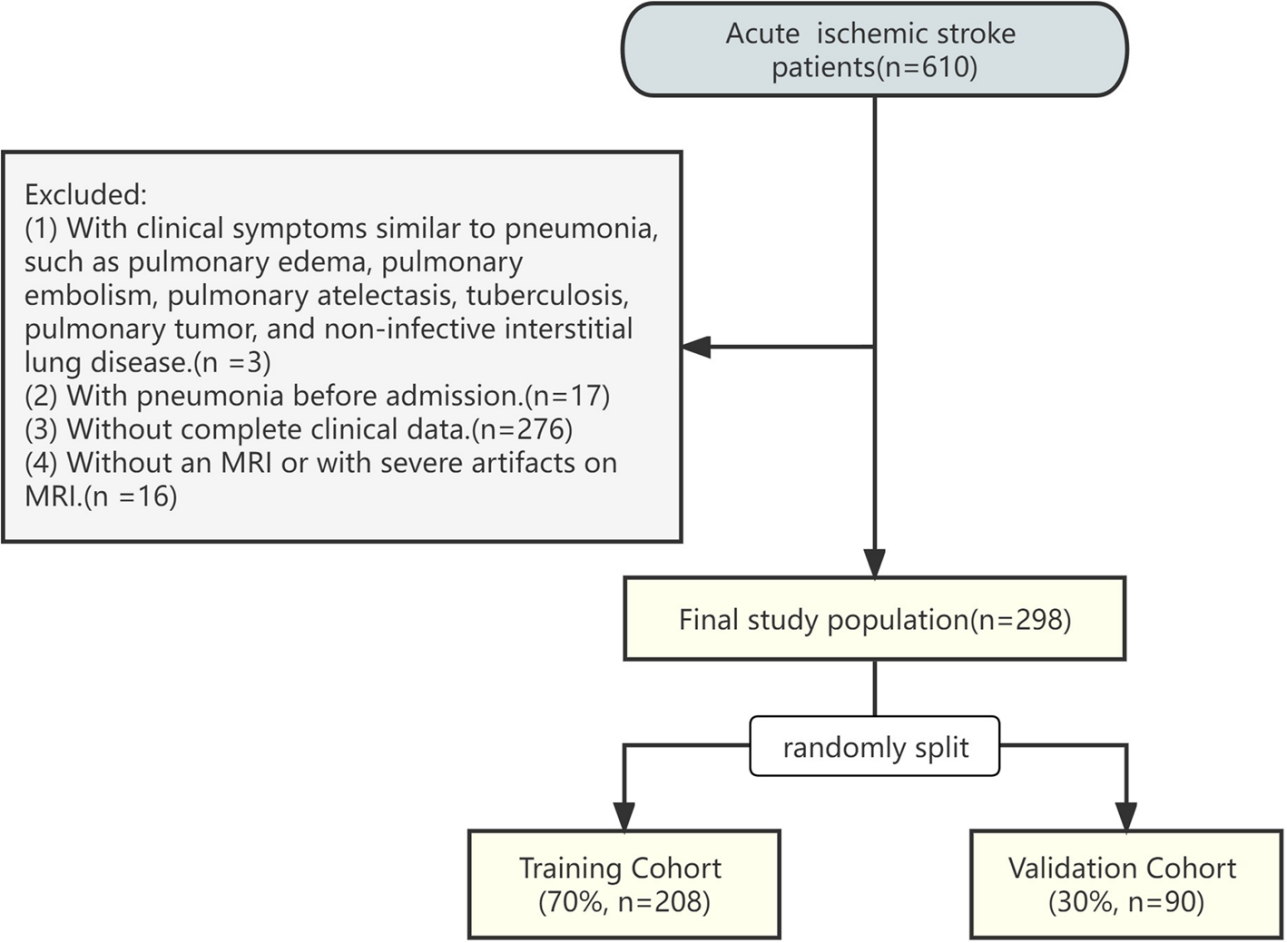
The flow chart of patients' enrollment

AIS patients who underwent a DWI-MRI scan from January 2018 to December 2021 were selected from our institution. All patients aged 18 years or above who were immediately hospitalized within 24 h following the onset of AIS symptoms and had a confirmed diagnosis of AIS on DW-MRI as defined by the World Health Organization [30, 31] and a National Institute of Health stroke scale (NIHSS) score of 15 or less were included in the study. Patients that presented with diseases that had clinical symptoms similar to pneumonia, such as pulmonary edema, pulmonary embolism, pulmonary atelectasis, tuberculosis, pulmonary tumor, and non-infective interstitial lung disease (*n* = 3), and those who had pneumonia before admission (*n* = 17) were excluded from the study. In addition, patients who lacked the complete clinical data (*n* = 276) and those who lacked an MRI or had severe artifacts on MRI (*n* = 16) were also excluded. The patients' enrollment flow chart was illustrated in Fig. 1. Finally, 298 patients were included. None of the patients underwent intravenous thrombolysis. The patients were randomly divided into a training cohort (*n* = 208) and a validation cohort (*n* = 90) at a ratio of 7:3.

### Diagnostic criteria for SAP

SAP was defined as a 'spectrum of lower respiratory tract infections occurring within the first 7 days after the onset of stroke'. Clinicians diagnosed SAP according to the data retrieved from chest images, clinical signs and symptoms, and laboratory parameters per guidelines issued by the Centers for Disease Control and Prevention criteria (CDC) [32]. The patients diagnosed with SAP were recorded and were divided into the SAP and non-SAP groups according to the criteria above.

### Ethical considerations

The study was approved by the Medical Ethics Committee of our institution: (2018) Medical Research Review No.04. Written informed consent was obtained from all patients participating in this study.

### Clinical data extraction

The clinical data, including demographics, past history, comorbidities, characteristics of condition on admission, laboratory results and image features, were extracted for the first time after admission and retrieved from the patient's medical records. The demographics included age, sex and body mass index (BMI). Past history included smoking and stroke. Comorbidities included hypertension, diabetes and dyslipidemia. Characteristics of condition on admission included dysphagia, NIHSS score, and the Modified Rankin Scale (mRS) score. The mRS score was used to measure the level of disability following AIS. Based on this score, the patients were divided into functionally independent if they had an mRS score of 2 or less and functionally dependent if they had an mRS score above 2 [33, 34]. Laboratory results included platelets (PLT), creatinine (Cr), aspartate transaminase to alanine transaminase ratio (AST/ALT), total cholesterol (Tch), low-density lipoprotein (LDL), high-density lipoprotein (HDL), triglycerides (TG), fasting plasma glucose (FPG), homocysteine (HCY), albumin (ALB), and the neutrophil to lymphocyte ratio (NLR). Image features included stenosis location (internal carotid artery (ICA), middle cerebral artery (MCA)), the extent of the stenosis (more or less than 50%), infarction side (left, right, or bilateral) and volume.

### MRI acquisition

All patients underwent a DWI-MRI within 24 h of admission. The DWI-MRIs were acquired using a GE 3.0 T MRI scanner, using a repetition time (TR) of 4090 ms, an echo time (TE) of 98.0 ms, a field of view (FOV) = 230 mm x 230 mm, a matrix of 192 × 192, a slice thickness to gap ration of 5 mm/1.5 mm, a b value of 0, and 1000 s/mm^2^.

### Lesion site annotating and feature extraction

**Fig. 2:**
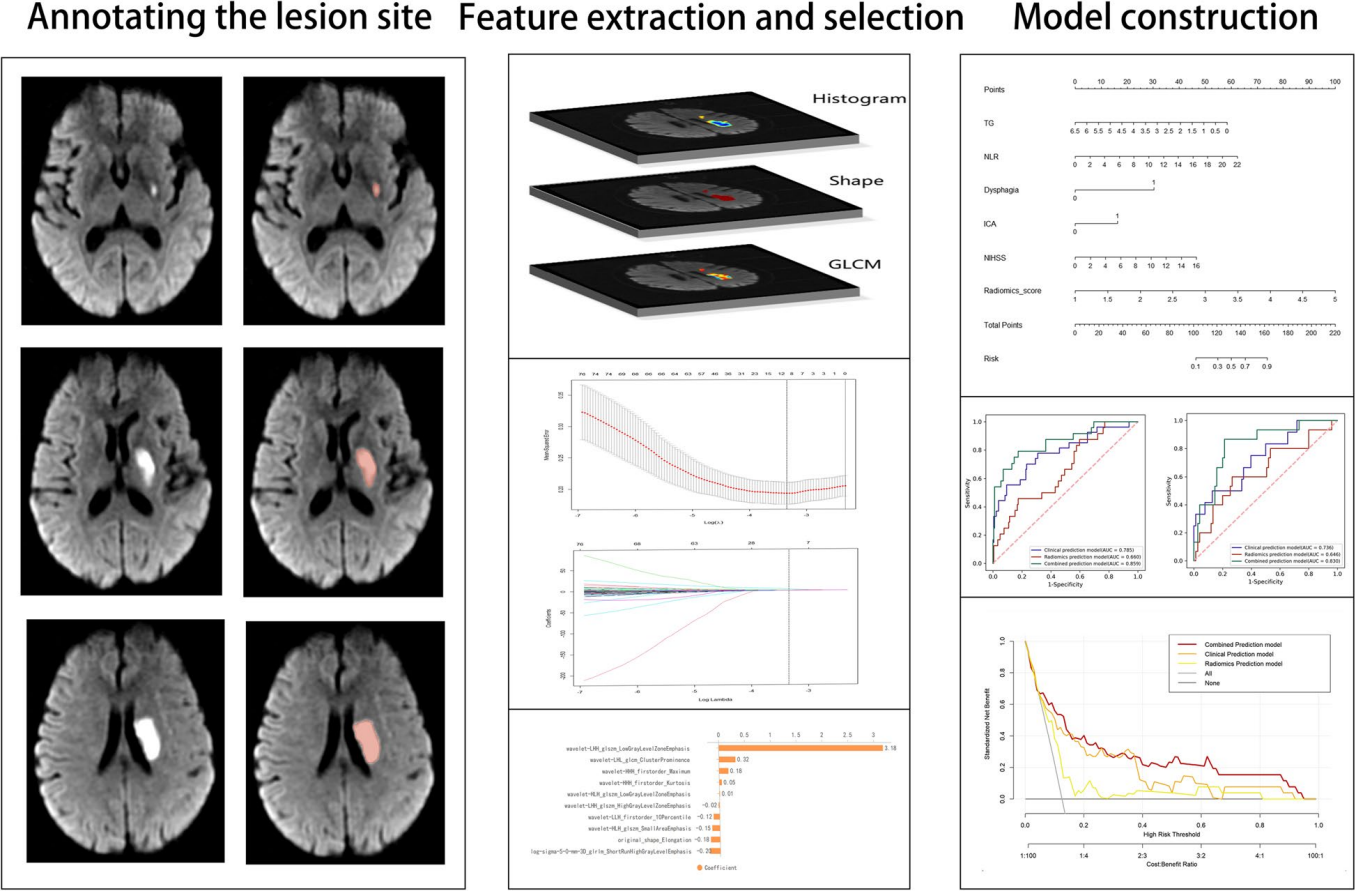
Flowchart of radiomics analysis

The radiomics analysis process was divided into 4 phases; lesion site annotation, feature extraction, feature selection, and model construction (Fig. 2). Two radiologists annotated the ischemic lesions on the patient’s DWI using the 3D-Slicer software version 4.10.2. Then, the consistency of the annotated volumes was evaluated by calculating the intra-class correlation coefficients (ICC). An ICC greater than 0.75 indicates a good agreement [35].

Subsequently, radiomics features were extracted using the PyRadiomics version 3.0.1 software as indicated by the Image Biomarker Standardization Initiative [36]. These features included first-order, shape and texture. The first-order and texture features were extracted from the original image, the Gaussian Laplace filtered image, and the wavelet filtered image.

### Feature selection and model construction

Univariate analysis was used to identify the factors that differed between all patients that developed SAP and those that did not. Then, the significant variables in the training cohort were inputted into the multivariate logistics regression (MLR) model to determine the independent clinical predictors of SAP (*P* < 0.05). These features were then used to construct the clinical prediction model. The risk ratios of the predicted factor were expressed as odds ratio (OR) (95% confidence interval).

Spearman's correlation coefficient was used to calculate the correlation and redundancy of features in the training cohort. The features were classified as redundant if they had a Spearman correlation coefficient higher than 0.8. For each pair of features that have been marked as redundant, choose to retain one of the features, the other redundant feature was removed [37]. Subsequently, the optimal predictive features were screened out utilizing the least absolute shrinkage and selection operator (LASSO) with ten-fold cross-validation [38]. Eventually, the radiomics score was calculated for each patient based on the linear combination of weighted selection parameters for the relevant LASSO coefficients of the optimal features, which was used to construct the radiomics prediction model. Finally, a combined prediction model was built based on both radiomics and clinical features, as well as a clinical prediction model containing only the clinical features and a radiomics prediction model containing only the radiomics features were built for comparison. Then, the best-performing model was developed into a nomogram. Subsequently, a subgroup analysis was performed based on age, sex, BMI, and stroke severity on admission to test the generalization ability of our model.

### Statistical analyses

The Python software (version 3.0) and R software (version 4.2.1) were used for statistical analyses. The normally distributed variables were expressed as mean ± standard deviation (SD), while the non-normally distributed variables were expressed as median (first quartile, third quartile). The variables between the SAP and non-SAP groups were compared using the student's t-test for the normally distributed variables and the Mann–Whitney U test for the non-normally distributed variables. The categorical variables were expressed as numbers (percentage), and the chi-squared (χ2) test was used for comparison. Pearson correlation analysis was used to test the correlation. The predictive performance of all 3 models was compared by calculating the area under the curve (AUC) of a receiver operating characteristics curve (ROC), the accuracy, sensitivity, specificity, negative predictive values (NPV), and positive predictive values (PPV). The Delong test was used to compare the differences in ROC curves between the models. The nomogram's accuracy was evaluated using calibration curves [39]. A decision curve analysis (DCA) was used to evaluate the clinical value of the nomogram in the validation cohort [40]. For all statistical tests, a p-value below 0.05 was considered statistically significant.

## Results

### Clinicodemographic features

The clinicodemographic features of the SAP and non-SAP patients in the training and validation cohorts are summarized in Table 1. A total of 298 patients were enrolled in this study, of whom 208 were assigned to the training cohort, and 90 were assigned to the validation cohort randomly. Of the 298 patients enrolled in the study, 39 (13.09%) patients developed SAP within 7 days following admission. A significant difference was noted in the age, BMI, dysphagia, NIHSS score, mRS score (> 2), TG, NLR, stenosis location (ICA) and volume between the SAP and non-SAP patients of the training cohort (all *P* < 0.05).

**Table 1:** Risk factors in the training and validation cohorts

**Characteristics**	Training Cohort (*n* = 208)			Validation Cohort (*n* = 90)		
	Without SAP (*n* = 180)	With SAP (*n* = 28)	*P*	Without SAP (*n* = 79)	With SAP (*n* = 11)	*P*
**Demographics**						
Age, M(Q25,Q75)	63.00(55.00,71.00)	68.50(60.00,76.00)	0.044	63.00(56.00,71.00)	66.00(61.00,77.00)	0.230
Sex, n(%)			0.250			0.957
Male	118(65.56)	22(78.57)		54(68.35)	8(72.73)	
Female	62(34.44)	6(21.43)		25(31.65)	3(27.27)	
BMI, M(Q25,Q75)	23.86(21.51,25.39)	21.34(20.20,24.61)	0.012	23.63(21.26,25.39)	22.46(20.76,23.66)	0.089
**Past History**						
Stroke, n(%)	54(30.0)	6(21.43)	0.480	22(27.85)	2(18.18)	0.752
Smoking, n(%)	62(34.44)	15(53.57)	0.082	24(30.38)	5(45.45)	0.510
**Comorbidities**						
Hypertension, n(%)	137(76.11)	19(67.86)	0.482	64(81.01)	8(72.73)	0.809
Diabetes, n(%)	68(37.78)	8(28.57)	0.465	34(43.04)	3(27.27)	0.504
Dyslipidemia, n(%)	43(23.89)	3(10.71)	0.188	16(20.25)	1(9.09)	0.635
**Characteristics of condition on admission**						
Dysphagia, n(%)	7(3.89)	8(28.57)	< 0.001	3(3.8)	4(36.36)	0.001
NIHSS score, M(Q25,Q75)	3.00(1.00,6.00)	4.00(2.00,10.00)	0.038	2.00(0.00,5.00)	5.00(2.00,12.00)	0.041
mRS score(> 2), n(%)	36(20.0)	13(46.43)	0.005	13(16.46)	4(36.36)	0.242
**Laboratory results**						
PLT, M(Q25,Q75)	206.50(174.00,240.00)	214.50(163.00,259.00)	0.829	199.00(164.00,227.00)	219.00(201.00,265.00)	0.061
Cr, M(Q25,Q75)	73.57(62.34,90.84)	72.35(65.55,82.69)	0.428	76.03(66.57,93.53)	78.33(59.64,85.72)	0.622
AST/ALT, M(Q25,Q75)	1.19(0.96,1.52)	1.36(1.09,1.55)	0.133	1.17(0.99,1.49)	1.31(0.95,1.44)	0.409
Tch, M(Q25,Q75)	4.67(4.04,5.20)	4.68(3.67,5.36)	0.518	4.67(4.16,5.19)	4.71(4.06,5.11)	0.735
LDL, M(Q25,Q75)	2.77(2.36,3.18)	2.73(2.02,3.39)	0.757	2.84(2.36,3.32)	2.92(2.42,3.39)	0.878
HDL, M(Q25,Q75)	1.07(0.93,1.31)	0.98(0.85,1.32)	0.360	1.02(0.92,1.29)	0.99(0.81,1.49)	0.907
TG, M(Q25,Q75)	1.43(1.11,2.03)	1.11(0.96,1.65)	0.019	1.59(1.17,2.02)	1.09(0.83,1.69)	0.036
FPG, M(Q25,Q75)	5.38(4.70,7.20)	5.42(4.42,5.79)	0.359	5.90(5.13,7.61)	5.44(5.11,6.16)	0.334
HCY, M(Q25,Q75)	14.16(11.54,18.14)	15.84(11.45,19.34)	0.361	14.54(11.68,18.12)	15.35(10.85,19.15)	0.956
ALB, M(Q25,Q75)	37.88(35.73,40.12)	37.02(33.86,38.22)	0.075	37.95(36.08,40.38)	36.35(35.07,38.16)	0.040
NLR, M(Q25,Q75)	2.65(1.91,3.62)	3.44(2.16,5.42)	0.008	2.61(2.05,3.70)	4.71(3.82,6.77)	< 0.001
**Imaging features**						
Stenosis location, n(%)						
ICA	19(10.56)	9(32.14)	0.007	8(10.13)	4(36.36)	0.054
MCA	42(23.33)	10(35.71)	0.241	21(26.58)	4(36.36)	0.749
Infarction side, n(%)						
Left	69(38.33)	7(25.00)	0.249	35(43.75)	3(27.27)	0.456
Right	75(41.67)	14(50.00)	0.533	29(36.25)	4(36.36)	0.755
Bilateral	31(17.22)	8(28.57)	0.242	20(25.32)	3(27.27)	0.818
Volume, M(Q25,Q75)	1.27(0.49,4.82)	4.08(0.85,16.07)	0.017	1.22(0.42,6.07)	1.84(0.40,16.18)	0.566

### Identification of the independent clinical predictors

The MLR identified TG, NLR, NIHSS score, dysphagia, and stenosis location (ICA) as independent predictors for SAP (Table 2). Based on the result of the MLR, the clinical prediction model was defined by the formula: Y = -1.059TG + 0.373NLR + 0.188NIHSS score + 2.433Dysphagia + 1.542stenosis location (ICA).

**Table 2:** Univariate and multivariable regression findings

Risk factor	β	SE	Wald	OR (95% CI)	*P*
TG	-1.059	0.485	4.77	0.347(0.134,0.897)	0.029
NLR	0.373	0.112	11.163	1.452(1.167,1.807)	0.001
NIHSS score	0.188	0.073	6.694	1.207(1.047,1.392)	0.01
Dysphagia	2.433	0.747	10.598	11.392(2.633,49.311)	0.001
Stenosis location (ICA)	1.542	0.633	5.939	4.674(1.352,16.155)	0.015

### The association between stenosis location (ICA), infarct volume and SAP

**Fig. 3:**
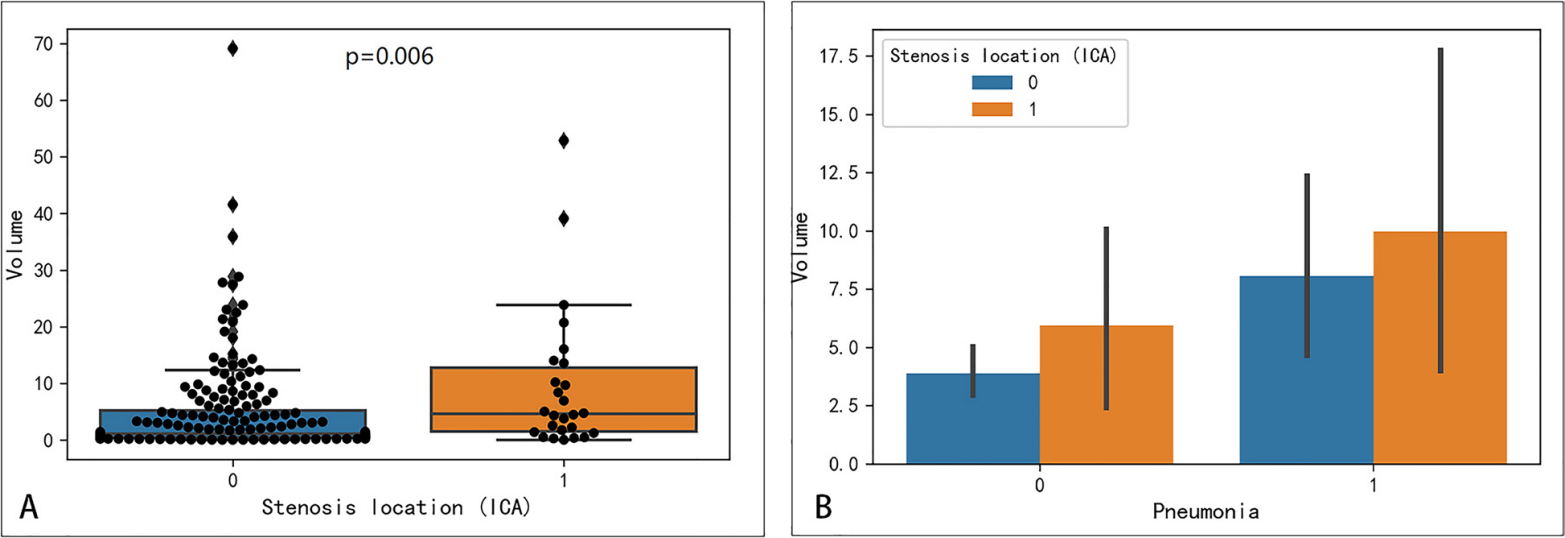
The association between stenosis location (ICA), infarct volume and SAP. **A** The distribution of infarct volume in patients with ICA stenosis and patients without ICA stenosis. **B** The distribution of infarct volume in patients with SAP and patients without SAP

The infarct volume was found to be significantly larger in patients with ICA stenosis than in patients without ICA stenosis (*P* = 0.006) (Fig. 3A).

The infarct volume was found to be significantly larger in patients with SAP than in patients without SAP (*P* = 0.017) (Table 1, Fig. 3B). In addition, both in patients with SAP and in patients without SAP, infarct volume was larger in patients with ICA stenosis.

**Fig. 4:**
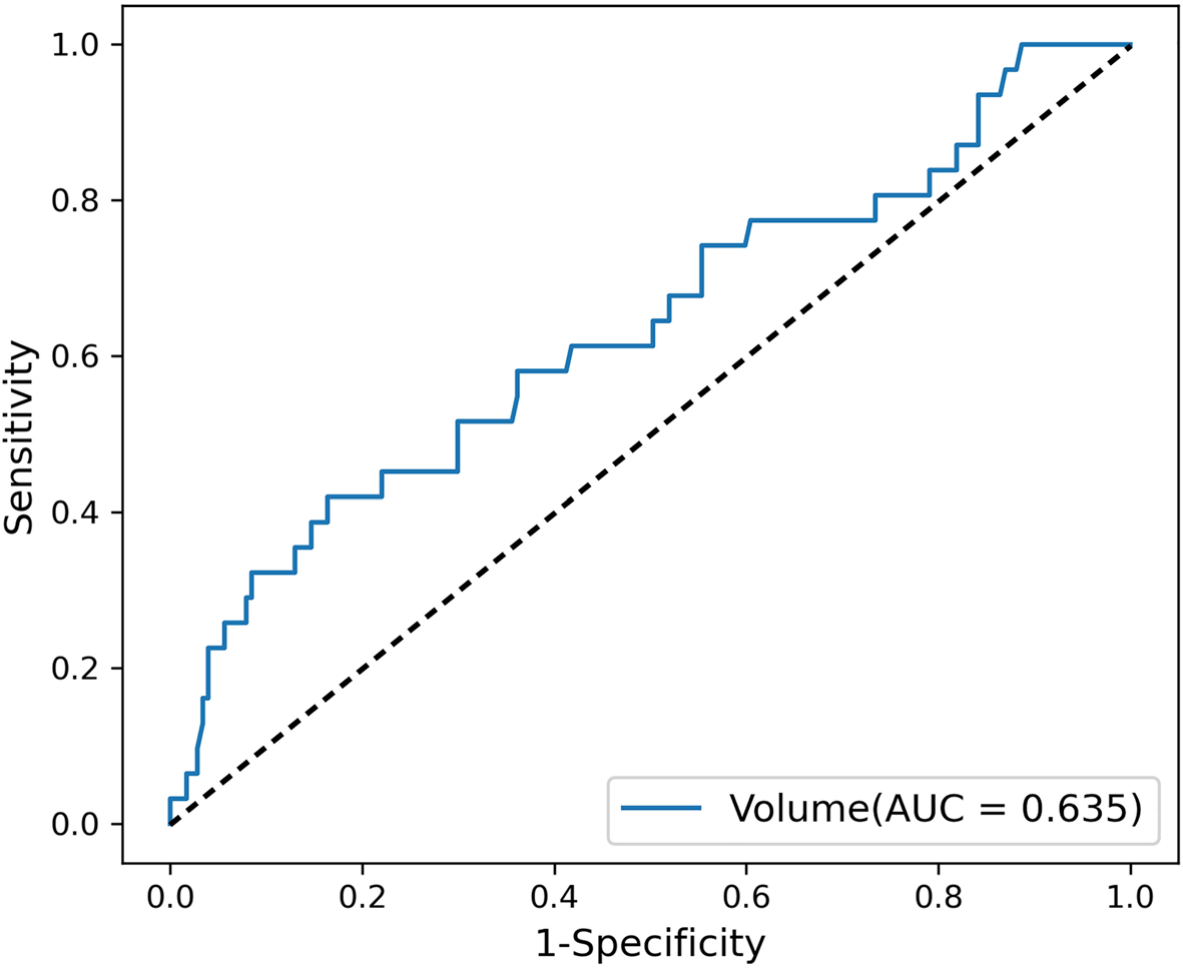
Performance of infarct volume in predicting SAP

Infarct volume showed moderate performance in predicting whether AIS patients would develop SAP, with an AUC of 0.635 (Fig. 4).

### Feature extraction and radiomics scores

**Fig. 5:**
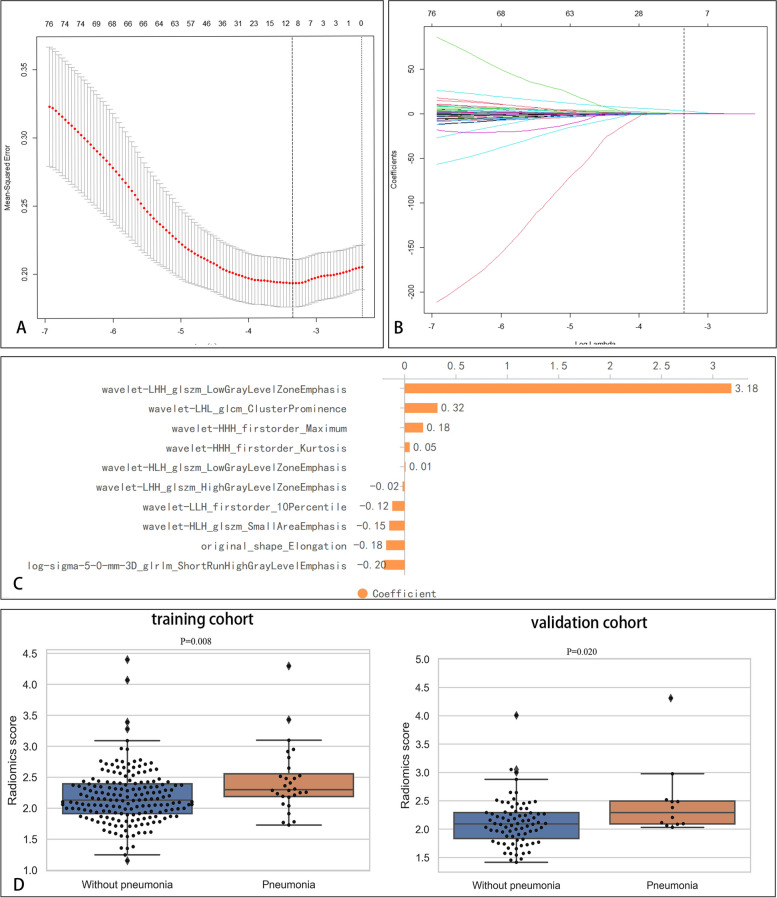
The optimal radiomics features for SAP. **A** The tuning parameter (k) in the tenfold cross-validation LASSO model. **B** The coefficients plotted against log(k). **C** The most relevant radiomics features predictive of SAP. **D** The distribution of radiomics scores in the training and validation cohorts

A total of 1041 features predictive of SAP were extracted. The 10 most relevant radiomics features for SAP in the training cohort were obtained by LASSO with ten-fold cross-validation (Fig. 5A-C). The distribution of the radiomics scores of patients with and without SAP for the training and validation cohorts is illustrated in Fig. 5D. The radiomics scores of patients with SAP and those without SAP were 2.30 (2.18, 2.65) and 2.13 (1.91, 2.40), respectively, for the training cohort and 2.29 (2.09, 2.52) and 2.09 (1.82, 2.29), respectively for the validation cohorts. The radiomics scores in patients with SAP were generally higher than in those without SAP. Wilcoxon's test showed a significant difference in the radiomics scores between the patients with SAP in both the training and validation cohorts (*P* < 0. 05). The mean ICC between the lesion volumes annotated by the 2 radiologists was 0.99 (95% CI 0.99–1, *P* < 0.05), indicating that the reproducibility of the feature extraction was good.

**Fig. 6:**
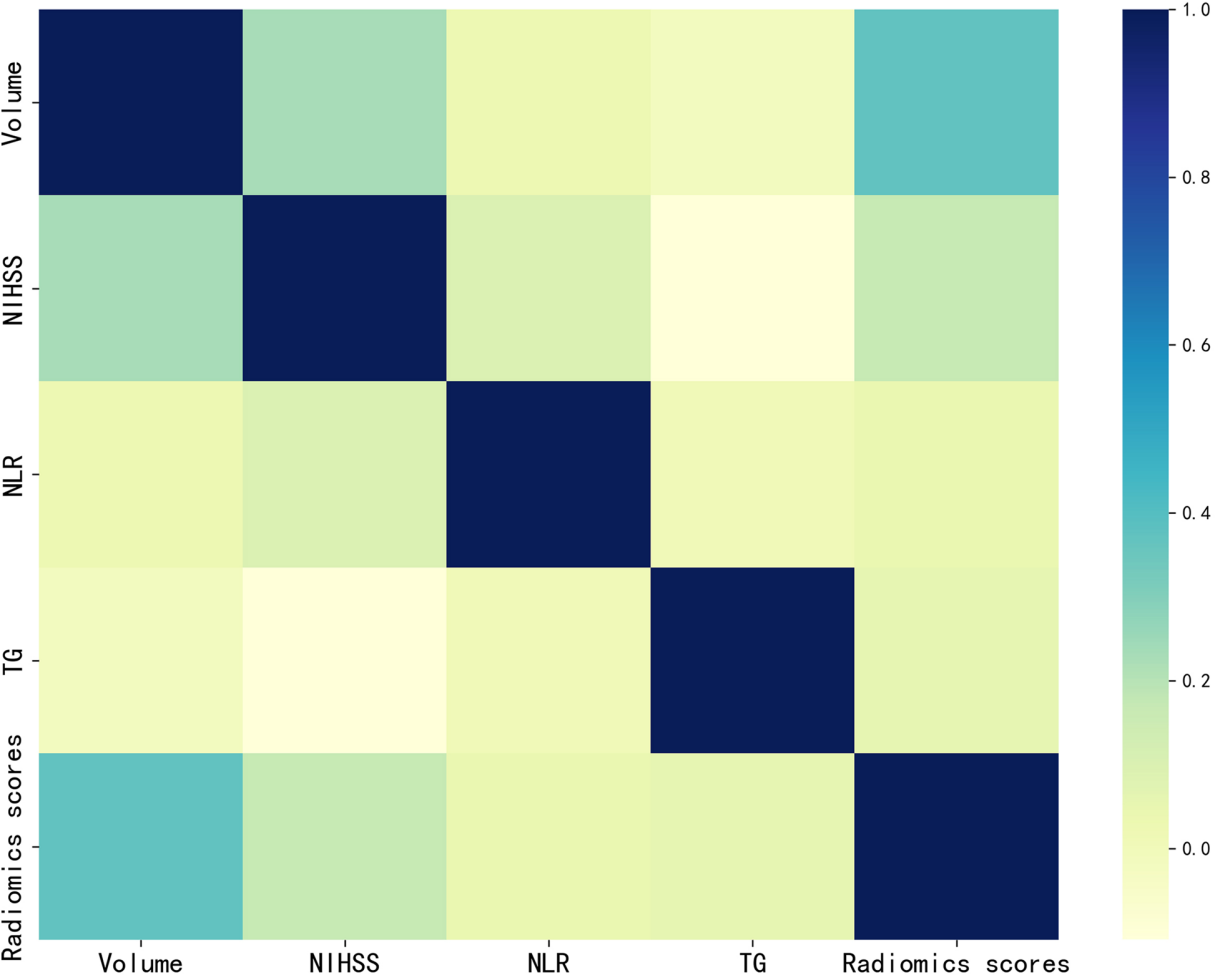
Correlation analysis chart

**Fig. 7:**
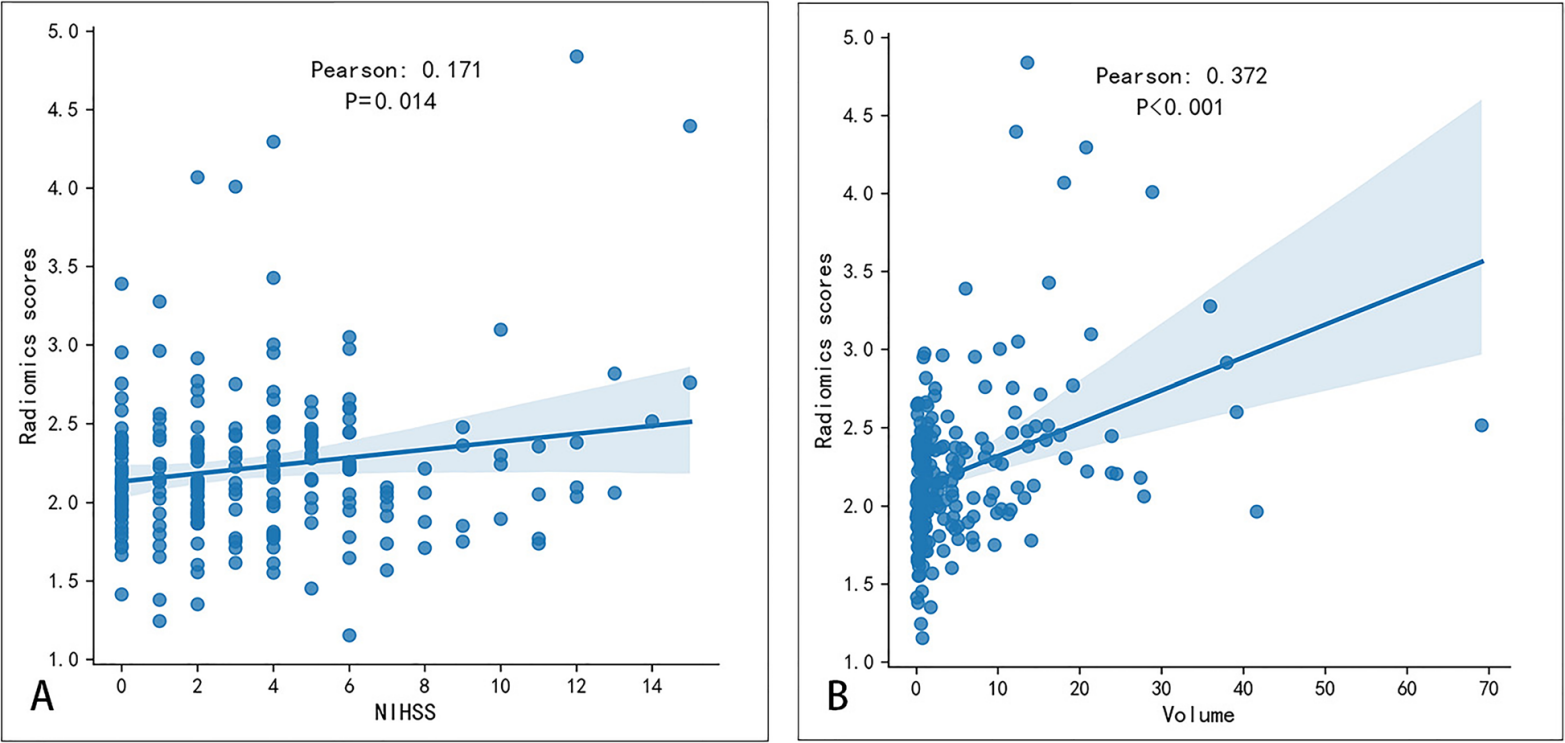
The association between stenosis location (ICA), infarct volume and SAP. **A** Association between radiomics scores and NIHSS scores. **B** Association between radiomics scores and volume

We investigated the association between radiomics scores and infarct volume as well as radiomics scores and independent risk factors (NIHSS, NLR, TG) (Fig. 6). The radiomics scores and NIHSS scores exhibited a positive linear relationship, with a Pearson correlation coefficient (r) of 0.171(*P* = 0.014) (Fig. 7A). The radiomics scores and volume exhibited a positive linear relationship, with a Pearson correlation coefficient (r) of 0.372(*P* < 0.001) (Fig. 7B).

### Prediction performance of the models

**Fig. 8:**
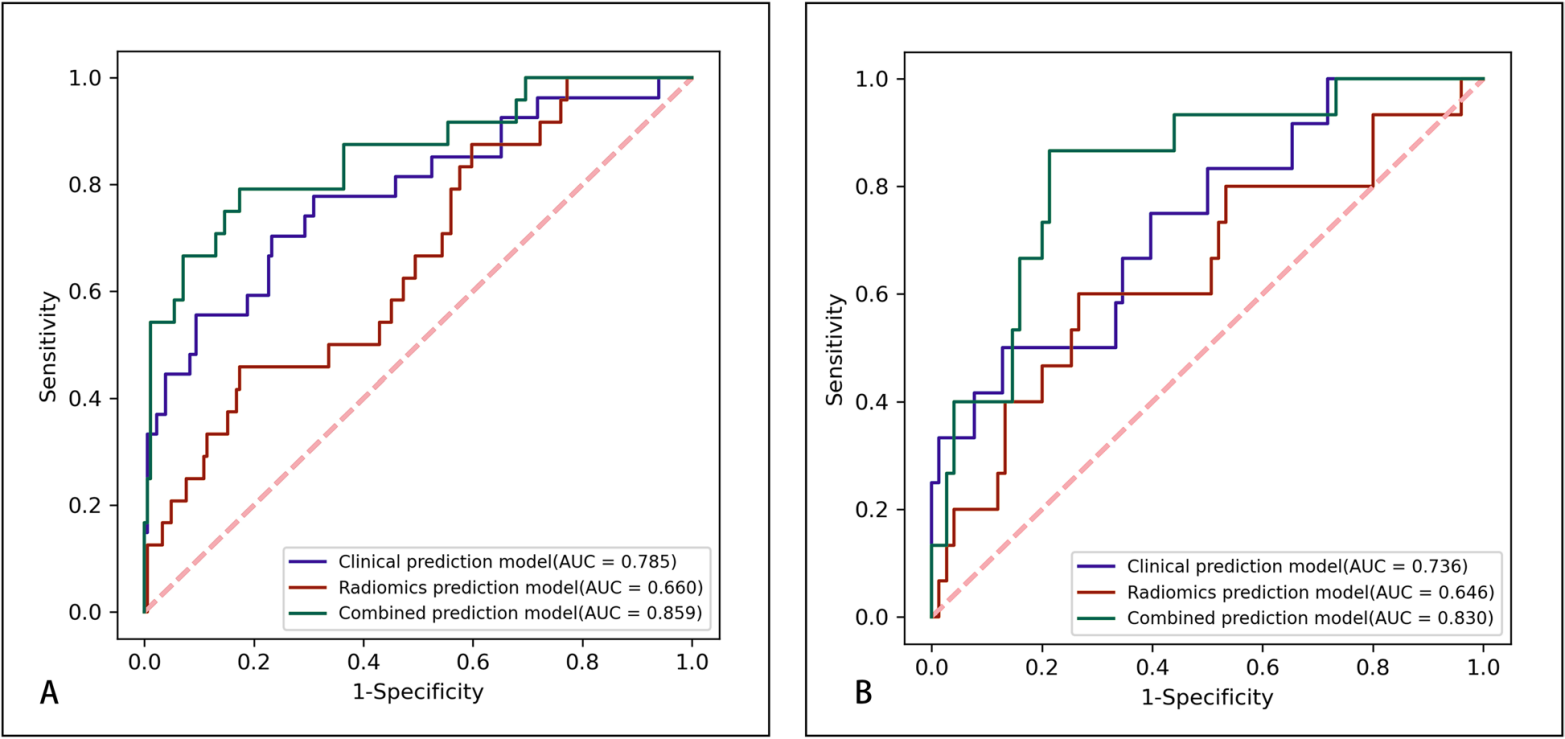
ROC curves of the 3 prediction SAP models for the training (**A**) and validation (**B**) cohorts

The prediction performance of the 3 models is summarized in Table 3, while Fig. 8 illustrates the ROCs for the 3 models. The clinical prediction model achieved an AUC of 0.785 (95%CI 0.673–0.889) and 0.736 (95%CI 0.629–0.837) for the training and validation cohorts, respectively. The sensitivity and specificity of the model were 0.667 and 0.768, respectively, in the training cohort and 0.417 and 0.872, respectively, in the validation cohort.

**Table 3:** The performance of three prediction models in the training and validation cohorts

Model	Cohort	AUC (95%CI)	ACC	Sensitivity	Specificity	NPV	PPV
Radiomics Prediction model	Training Cohort	0.660(0.546–0.766)	0.779	0.417	0.826	0.916	0.238
	Validation Cohort	0.646(0.541–0.756)	0.700	0.533	0.733	0.887	0.286
Clinical Prediction model	Training Cohort	0.785(0.673–0.889)	0.759	0.667	0.768	0.939	0.300
	Validation Cohort	0.736(0.629–0.837)	0.811	0.417	0.872	0.907	0.333
Combined Prediction model	Training Cohort	0.859(0.759–0.936)	0.817	0.750	0.826	0.962	0.360
	Validation Cohort	0.830(0.758–0.896)	0.789	0.800	0.787	0.952	0.429

The radiomics prediction model achieved an AUC of 0.660 (95% CI 0.546–0.766) and 0.646 (95% CI 0.541–0.756) in the training and validation cohorts, respectively. The sensitivity and specificity of the model were 0.417 and 0.826, respectively, in the training cohort and 0.533 and 0.733, respectively, in the validation cohort.

The combined prediction model had an AUC of 0.859 (95% CI 0.759–0.936) and 0.830 (95% CI 0.758–0.896) in the training and validation cohorts, respectively. The sensitivity and specificity of the model were 0.750 and 0.826, respectively, in the training cohort and 0.800 and 0.787, respectively, in the validation cohort.

Compared with the other 2 models, the combined model had a significantly higher AUC in both the training and validation cohorts (Delong test *P* < 0.05) (Table 4) and was therefore used to develop the clinical nomogram.

**Table 4:** Comparison of ROC between the models using the Delong test

Cohort	The models for comparison	*P*
Training Cohort	Combined Prediction model versus Clinical Prediction model	0.042
	Combined Prediction model versus Radiomics Prediction model	0.006
Validation Cohort	Combined Prediction model versus Clinical Prediction model	0.043
	Combined Prediction model versus Radiomics Prediction model	0.05

### Development and validation of the nomogram 

**Fig. 9:**
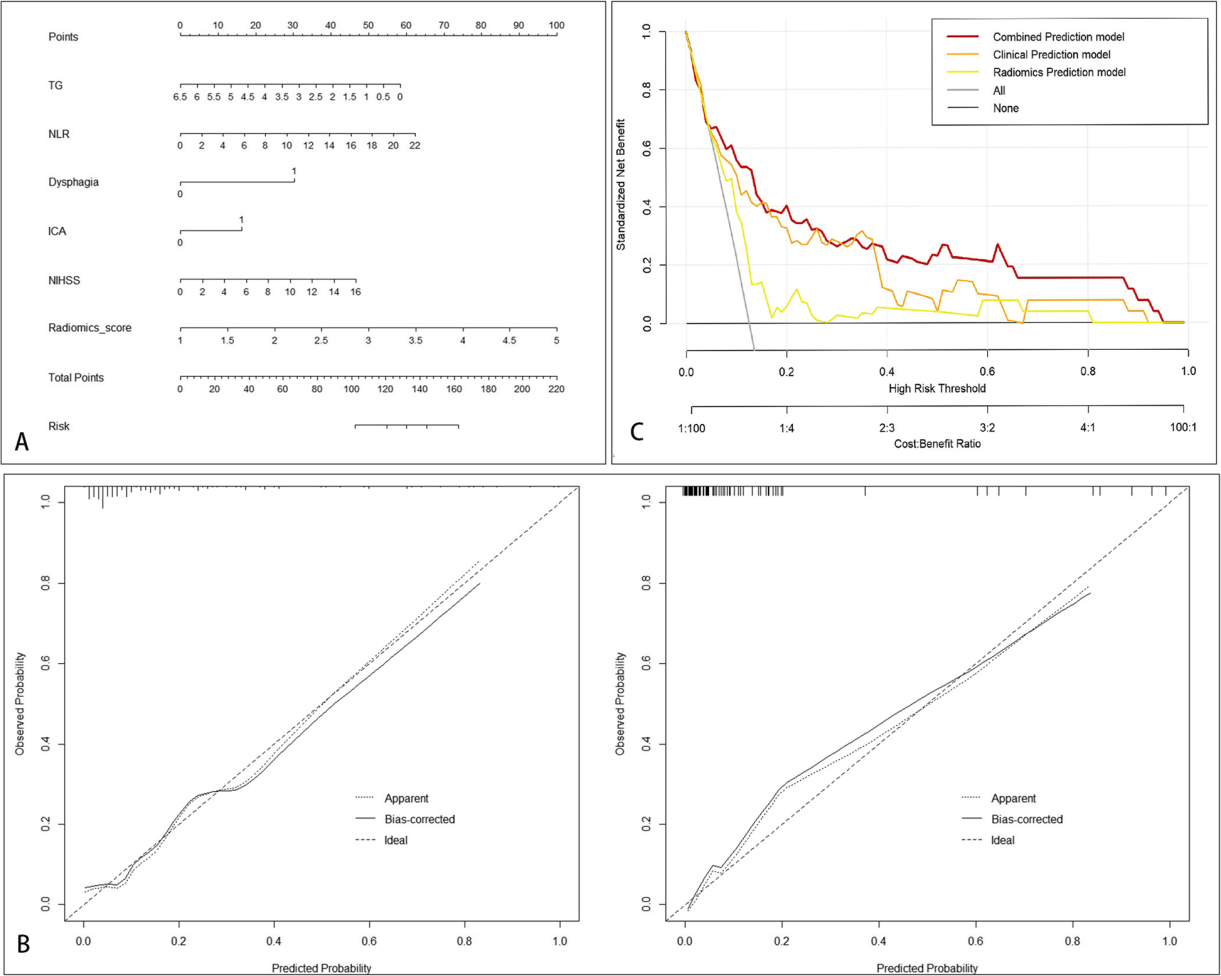
Nomogram of the combined prediction SAP model. **A** The nomogram. **B** The calibration curves for the training and validation nomograms. **C** The decision curve for the nomogram

The nomogram of the combined model is illustrated in Fig. 9A. Clinicians could use the nomogram to predict the risk of developing SAP by summing the risk of the relevant clinical variables and the radiomics risk score. The calibration curves for the training and validation nomograms are illustrated in Fig. 9B. The calibration plot displayed a good level of consistency between the predicted and actual probabilities for both cohorts. The nomogram's DCA is displayed in Fig. 9C. The DCA confirmed the clinical utility of the model.

**Fig. 10:**
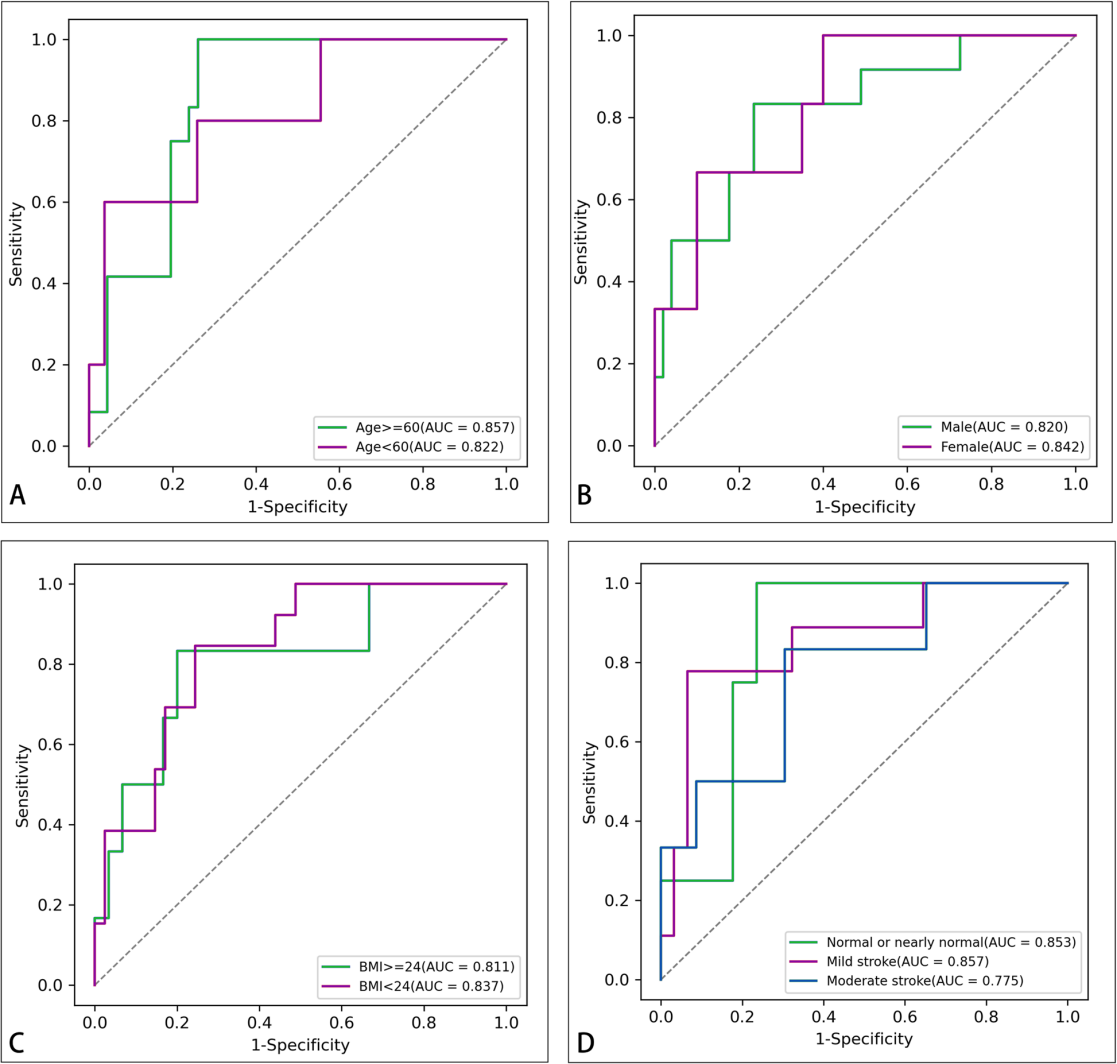
Subgroup analysis of the combined model. After dividing by the age (**A**), sex (**B**), BMI (**C**), and stroke severity on admission (**D**)

As shown in Table 5 and Fig. 10, the subgroup analysis showed that the performance of the nomogram was not influenced by patient age, sex, BMI and stroke severity on admission (Delong test *P* > 0.05).

**Table 5:** Subgroup analysis of AUCs using the Delong test

Subgroups divided by	AUC	*P* (versus overall set)
Age		
Age > = 60	0.857	0.694
Age < 60	0.822	0.927
Sex		
Male	0.820	0.914
Female	0.842	0.928
BMI		
BMI > = 24	0.811	0.878
BMI < 24	0.837	0.942
Stroke severity on admission		
Normal or nearly normal (NIHSS score 0–1)	0.853	0.804
Mild stroke (NIHSS score 2–4)	0.857	0.460
Moderate stroke (NIHSS score 4–15)	0.775	0.581

## Discussion

SAP is a potentially preventable complication of stroke. The early identification of SAP is essential to limit the adverse clinical outcome of pneumonia [41, 42]. In this study, we explored the predictive value of radiomics in predicting SAP and construct an artificial intelligent model based on clinical features and DWI-MRI radiomics features to predict SAP following AIS. The combined model performed better than clinical model and radiomics model. The DCA confirmed the clinical effectiveness of the proposed model.

It has been confirmed by several studies that brain MRI-based imaging features are closely related to SAP. Zhao et al. [43] showed that the DWI Alberta Stroke Program Early Computed Tomography Score (DWI-ASPECTS) used to predict the severity of AIS could also be used to predict the occurrence of SAP in patients with mild AIS [1, 44]. We investigated the association between radiomics scores, infarct volumes and NIHSS scores, found that there was a linear positive correlation between radiomics scores and NIHSS scores (Pearson:0.171, *P* = 0.014), as well as between radiomics scores and infarct volume (Pearson:0.372, *P* < 0.001). Yu et al. [17] found that neuroimaging features play a key role in predicting SAP. Brain atrophy and core infarct volume are closely related to the occurrence of SAP [45, 46]. We found that larger infarct volume and higher radiomics score were associated with a greater risk of developing pneumonia. We also investigated the predictive performance of infarct volume and obtained moderate performance, with an AUC of 0.635. The NIHSS score reflects the severity of the stroke [47]. Previous studies have shown that stroke severity increases the risk of developing SAP [1]. Therefore, the radiomics score reflects the severity of stroke to a certain extent and affects the occurrence of SAP.

Studies have shown a good association between several radiomics features and clinical stroke outcomes, such as prognosis and recurrence [18]. Currently, the radiomics features has been certified to improve the prediction ability of prognosis prediction. Tang et al. [48] found an association between specific morphology radiomics features and stroke recurrence in patients with symptomatic intracranial atherosclerotic stenosis. With the addition of radiomics features, the AUC of the prediction model was increased by 11.7% and 17% in the training and validation sets, respectively. Zhou et al. [49] demonstrated that the radiomics features performed well in predicting AIS outcomes. With the addition of radiomics features, the AUC of the prediction model was increased by 10.1% and 10.6% in the training and validation sets, respectively. However, relatively few studies have used radiomics features to predict the risk of complications following AIS. Immunological changes are associated with an increased tendency to respiratory infections [13]. And neuroanatomical correlates are associated with immunological changes after stroke and increased risk of infection, so it is easy to develop SAP [16]. An activation of the sympathetic nervous system is the main immunosuppressive mechanism leading to a high incidence of infections after stroke [50]. Studies have revealed that significant correlations with texture features and neural density in the side of the hippocampus contralateral to the ischemic area. These preliminary results suggest that texture features can reflect microscopic changes that occur post-stroke, even in an area spared by ischemia [29]. Therefore, we believe that the extracted radiomics features, belonging to the texture features, can reflect the microscopic changes that occur after a stroke and provide a good representation of neural alterations caused by low immunity. It may potentially be used to predict the risk of developing infections after AIS. In this study, we used the PyRadiomics software version 3.0.1 to extract radiomics features from the manually segmented brain lesions [18]. A total of 1041 radiomics features were obtained, of which 10 were identified as highly predictive of SAP. The majority of these features belonged to the wavelet feature cluster. The wavelet feature cluster measures asymmetry around the mean, which represents the tissue damage caused by the infarct. A study reported that this type of features might be associated with complications due to other pathological changes [49]. Among these features, LowGrayLevelZoneEmphasis is a feature used to describe the distribution of gray levels in an image, and in MRI images of stroke patients, lesion site usually shows different gray levels, which reflects the difference in density between the lesion site and the surrounding normal site. Higher LowGrayLevelZoneEmphasis values indicate that areas of lower gray levels are more prominent around the lesion site, which may suggest pathological changes in the contours or margins of the infarcts, reflecting heterogeneity of the lesion site, which may be associated with SAP. Cluster Prominence represents the cluster significance and is a measure of GLCM skewness and asymmetry, which was associated with stroke prognosis in previous studies [51], may be related to the occurrence of pneumonia. However, more research is required to understand the molecular mechanisms involved behind the development of this feature.

Consistent with previous studies, NLR, dysphagia, and NIHSS score were identified as independent predictors of SAP [1, 42, 52–54]. In addition, the stenosis location (ICA) and low TG were also identified as independent risk factors for predicting SAP. Chlamydia pneumoniae infection can also promote the development of atherosclerosis [55], Cao J et al. found that 84.0% of Chinese patients with carotid atherosclerotic plaques tested positive for chlamydia pneumonia-specific antigens [56]. As atherosclerotic plaques grow larger, leading to the narrowing of the arteries [57], the body's immunity system is weakened, leading to an increased risk of infections [16]. A previous study reported that when compared to MCA, the ICA causes larger infarcts, thus increasing the risk of SAP [58]. Our study found that patients with ICA stenosis were more likely to develop SAP, and this relationship persisted after adjustment for confounding factors. Patients with ICA stenosis had larger infarct volumes, and larger infarct volumes can affect multiple brain functions and impair immunity, ultimately leading to functional impairment and increased susceptibility to infections [16]. TGs are a potential source of arachidonic acids. The lipases and cyclooxygenase found in the lipid droplets of macrophages can catalyze the esterification of arachidonic acid to gradually transform it into eicosanoids compounds [59, 60]. These compounds are important lipid mediators of inflammation, and have an important role in balancing the extent of the inflammatory response [61]. Some studies have shown that when the concentration of TG is high, the TG-rich lipoprotein can combine with lipopolysaccharide to exert immune regulation on cells crucial for hosting the immune defense [62], thus reducing the risk of SAP. The same association was not reported in other studies [63, 64]. Therefore, further research is recommended to confirm the specific relationship between TG and SAP.

Our study has some limitations that have to be acknowledged. Since this study was based on data extracted retrospectively, the model's prediction accuracy needs to be validated prospectively. The use of single-agency data may lead to issues of geographic specificity and representativeness of the sample, a limitation that may restrict our generalizations about different groups. Therefore, in future studies, we will actively explore the possibility of external validation to enhance the reliability and generalizability of the study. In addition, the effect of treatment during hospitalization was not taken into account in this model. Finally, the combined model had a high NPV and a low PPV, possibly due to the low incidence of SAP in our cohort (SAP: 39/298, 13.1%).

## Conclusion

The addition of the radiomics features to the clinical model improved the prediction of SAP in AIS patients, which verified its feasibility. The proposed nomogram could identify patients at risk of developing SAP and thus provide timely interventions.

## Data Availability

The data that support the findings of this study are available on request from the corresponding author. The data are not publicly available due to privacy or ethical restrictions.
